# Immune-Related Thyroid Dysfunction in PD-L1 High Non-Oncogene-Addicted NSCLC Treated with First-Line Pembrolizumab: Incidence, Timing, and Predictive Impact

**DOI:** 10.3390/curroncol33020109

**Published:** 2026-02-12

**Authors:** Filip Marković, Mihailo Stjepanović, Milica Kontić

**Affiliations:** 1 Clinic for Pulmonology, University Clinical Centre of Serbia, 11000 Belgrade, Serbia; 2 Faculty of Medicine, University of Belgrade, 11000 Belgrade, Serbia

**Keywords:** immune related adverse events, immune check-point inhibitors, non-small cell lung cancer, pembrolizumab, thyroid

## Abstract

In metastatic NSCLC with high PD-L1 expression (TPS ≥ 50%), pembrolizumab monotherapy yields durable benefit in a subset of patients. Immune-related thyroid dysfunction (irTD) is common during PD-1/PD-L1 blockade, but its predictive value remains uncertain. We conducted a retrospective, single-center study including 363 patients with metastatic NSCLC, PD-L1 TPS ≥ 50%, and no actionable oncogenic drivers treated with first-line pembrolizumab. Thyroid function tests were performed at baseline and every six weeks. irTD was defined based on laboratory abnormalities with or without clinical symptoms and classified as early onset (≤90 days) or late onset (>90 days). Progression-free survival (PFS) was estimated using Kaplan–Meier methods and compared using log-rank tests. Cox proportional hazards models included irTD as a time-varying covariate. Landmark analyses at 3 and 6 months reduced immortal-time bias. Events were graded according to CTCAE v5.0. Among 363 eligible patients, irTD occurred in 110 (30.3%); median onset was 114 days (range 21–550). Median cohort PFS was 9.8 months (95% CI 7.26–12.34). Patients with irTD had significantly longer PFS than those without irTD: 26.33 (95% CI 19.09–33.57) vs. 6.16 months (95% CI 4.70–7.63), with an HR of 0.378 (95% CI 0.280–0.511; *p* < 0.001). Landmark analyses confirmed benefit at 3 months (28.4 vs. 13.7 months; HR 0.490, *p* < 0.001) and 6 months (29.0 vs. 20.5 months; HR 0.587, *p* < 0.001). PFS did not differ by irTD timing (early vs. late; HR 0.926, *p* = 0.682). Poor ECOG PS (≥2) was associated with worse outcomes and a lower incidence of irTD. We found that irTD is common, clinically manageable, and strongly associated with improved PFS in PD-L1-high metastatic NSCLC treated with pembrolizumab. Thyroid dysfunction may serve as a feasible on-treatment biomarker of effective immune activation, warranting further prospective validation.

## Simple Summary

In metastatic NSCLC with high PD-L1 expression, about one-third of patients treated with pembrolizumab develop immune-related thyroid dysfunction (irTD). Our study of 363 patients shows that those who experience irTD have significantly longer progression-free survival compared to those who do not. The timing of thyroid dysfunction (early or late) did not affect outcomes. Importantly, irTD was manageable and occurred more often in patients with good performance status. These results suggest that irTD may serve as a useful marker of effective immune response during pembrolizumab therapy and could help identify patients likely to derive long-term benefit.

## 1. Introduction

Lung cancer remains a critical global health concern, as it is the leading cause of cancer-related mortality worldwide [1]. Despite advances in detection and treatment, lung cancer continues to be associated with an unfavorable prognosis, particularly when diagnosed at later stages [1]. Non-small-cell lung cancer (NSCLC) represents the predominant histological subtype, accounting for roughly 80–85% of all lung cancer cases. Unfortunately, most patients are diagnosed with locally advanced or metastatic disease, limiting curative treatment options [2,3]. For many years, systemic cytotoxic chemotherapy constituted the backbone of treatment for advanced NSCLC, though it provided only modest survival benefits and was often associated with substantial toxicity.

Over the past decade, the therapeutic paradigm for NSCLC has shifted substantially with the development of immune checkpoint inhibitors (ICIs). These agents enhance antitumor immune responses by disrupting key mechanisms of immune escape, most notably signaling through the programmed death-1 (PD-1) and programmed death-ligand 1 (PD-L1) pathway. As a result, ICIs have demonstrated meaningful survival benefits in selected patient subgroups. Pembrolizumab, a humanized monoclonal antibody directed against PD-1, became the first immunotherapy approved for first-line monotherapy in patients with advanced NSCLC characterized by high PD-L1 expression (tumor proportion score [TPS] ≥ 50%) and absence of targetable oncogenic alterations, following the pivotal results of the KEYNOTE-024 trial [4,5]. However, more than half of these patients fail to achieve long-term clinical benefit from pembrolizumab monotherapy, emphasizing the need for better predictive biomarkers [6,7,8].

While immune checkpoints are crucial for maintaining self-tolerance, their inhibition can lead to immune-related adverse events (irAEs), which may affect virtually any organ system [9]. The most commonly involved organs include the skin, gastrointestinal tract, liver, lungs, and endocrine glands [9]. Notably, the development of irAEs has been associated with favorable clinical outcomes in some patients with advanced NSCLC receiving ICIs [10,11]. Among these irAEs, immune-related thyroid dysfunction (irTD) is one of the most frequently observed [9,10]. However, its predictive value in NSCLC remains unclear. Some studies have linked irTD to improved progression-free survival (PFS), while others have reported no significant impact [12,13,14]. Nevertheless, existing evidence remains heterogeneous, with many reports limited by small sample size, mixed treatment settings, inclusion of heterogeneous PD-L1 subgroups, or insufficient adjustment for immortal-time bias.

Importantly, data focusing exclusively on patients with PD-L1–high (TPS ≥50%), non-oncogene-addicted metastatic NSCLC treated uniformly with first-line pembrolizumab are scarce. Moreover, despite its prevalence, data on the incidence, characteristics, and clinical relevance of irTD—particularly in patients treated with pembrolizumab—remain limited. A clearer understanding of the timing, presentation, and potential prognostic implications of thyroid irAEs is essential for optimizing treatment monitoring and management in this setting.

This study aims to evaluate the incidence, clinical features, timing of onset, and predictive significance of irTD in patients with metastatic, non-oncogene-addicted NSCLC and PD-L1 TPS > 50% receiving first-line pembrolizumab therapy.

## 2. Materials and Methods

The study included patients with histologically confirmed metastatic NSCLC with PD-L1 TPS ≥ 50% that started treatment with pembrolizumab monotherapy between November of 2021 and April of 2024.

This retrospective analysis was performed at a single tertiary academic center in Serbia, using data derived from a hospital-based lung cancer registry. Prior to initiation of first-line systemic therapy, all patients underwent routine assessment of PD-L1 expression on formalin-fixed, paraffin-embedded tissue or cytological specimens. PD-L1 testing was conducted using the 22C3 monoclonal antibody clone (DAKO, Glostrup, Denmark), in accordance with standard clinical practice.

Molecular profiling before treatment initiation included testing for epidermal growth factor receptor (EGFR) mutations using the Cobas^®^ EGFR Mutation Test v2, (Roche Molecular Systems, Inc., Pleasanton, CA, USA) as well as evaluation of anaplastic lymphoma kinase (ALK) rearrangements by immunohistochemistry. Patients in whom actionable driver alterations were identified were excluded from the analysis; consequently, no patient included in the final cohort harbored a known oncogenic driver mutation.

Clinical follow-up was performed in line with routine institutional protocols, and treatment response was evaluated based on the Response Evaluation Criteria in Solid Tumors (RECIST), version 1.1 [15]. Treatment-related adverse events were graded using the Common Terminology Criteria for Adverse Events (CTCAE), version 5.0 [16].

### 2.1. Data

Clinical data were extracted from an institutional lung cancer registry that systematically records demographic information, clinical characteristics, pathological and molecular findings, as well as treatment details and survival outcomes of patients managed at our center. All data used for the present analysis were anonymized prior to evaluation.

This was a retrospective, observational real-world study, and all diagnostic, treatment, and follow-up procedures—including radiological restaging and response assessment according to RECIST version 1.1—were performed as part of routine clinical practice rather than within a prospective trial protocol.

### 2.2. Ethics Approval

The study was performed in accordance with the Declaration of Helsinki and approved by the Ethics Committee of the University Clinical Centre of Serbia (2268/4; 4 December 2025).

### 2.3. Definition and Management of Thyroid Dysfunction

Thyroid-related adverse events were evaluated using serial thyroid function testing in conjunction with clinical assessment. Hypothyroidism was defined by a serum thyroid-stimulating hormone (TSH) level exceeding 4.0 mIU/L in the presence of normal or decreased free thyroxine (FT4) concentrations. Hyperthyroidism was defined as a suppressed TSH level below 0.4 mIU/L accompanied by elevated FT4 and/or free triiodothyronine (FT3) levels. Thyroiditis was characterized by a transient hyperthyroid phase followed by subsequent development of hypothyroidism, consistent with a painless thyroiditis pattern. Thyroid function tests were performed at baseline, and patients with registered thyroid dysfunction were excluded from the study. Additionally patients that were previously diagnosed with a thyroid dysfunction were also excluded from the study. In addition to baseline testing, TFTs were performed every six weeks during pembrolizumab treatment, with additional assessments conducted as clinically indicated.

Early-onset irTD was defined as occurring within the first 90 days of ICI therapy, while irTD developing after 90 days was considered late-onset, in accordance with the Society for Immunotherapy of Cancer (SITC) consensus definition [17].

Hypothyroidism was managed with levothyroxine (typically initiated at 25–50 μg/day). Hyperthyroidism was treated with beta-blockers and, when needed, antithyroid medications. All treatments followed European Thyroid Association (ETA) guidelines [18].

Patients developing thyroid dysfunction were initially managed by the treating oncologist, with referral to an endocrinologist when clinically indicated.

### 2.4. Statistics

Descriptive statistics were used to summarize patient demographics, with baseline characteristics reported as counts and percentages. Median progression-free survival (PFS) was defined as the time from therapy initiation to disease progression, with censoring at the last follow-up for patients still alive without disease progression. PFS was estimated using the Kaplan–Meier method and compared using the log-rank test.

Thyroid irAEs were treated as a time-varying covariate, as their onset occurred at unspecified points during follow-up. To minimize immortal time bias (ITB), landmark Cox regression analyses were performed. Landmark time points were set at 3 and 6 months after ICI initiation, including only patients who were progression-free at those times.

Time-to-event outcomes were analyzed using Cox proportional hazards regression, with results expressed as hazard ratios (HRs) and corresponding 95% confidence intervals (CIs). The univariable models evaluated the following covariates: age (<70 vs. ≥70 years), sex, histological subtype (non-squamous vs. squamous), smoking history (current or former vs. never), PD-L1 tumor proportion score (50–80% vs. ≥80%), Eastern Cooperative Oncology Group performance status (0–1 vs. ≥2), presence of brain metastases, exposure to radiotherapy, and occurrence of thyroid immune-related adverse events. Variables demonstrating an association with outcomes at a significance level of *p* < 0.10 in univariable analyses were subsequently entered into the multivariable model.

The relationship between thyroid immune-related adverse events and treatment response was examined using the chi-square test. All statistical tests were two-tailed. Data analyses were conducted using SPSS software, version 23 (IBM Corp., Armonk, NY, USA).

## 3. Results

A total of 363 patients with metastatic non-small-cell lung cancer and high PD-L1 expression (tumor proportion score ≥ 50%), without evidence of actionable oncogenic alterations, received pembrolizumab monotherapy in the first-line setting. The cohort had a mean age of 65.77 years (range, 40–86), and men accounted for 59.5% of the study population. A history of tobacco exposure was common, with 63.9% of patients identified as current smokers and an additional 23.1% as former smokers. With respect to tumor histology, adenocarcinoma was the predominant subtype (68.0%), followed by squamous cell carcinoma (23.6%). Notably, 97 patients (26.7%) presented with impaired functional status, defined as an Eastern Cooperative Oncology Group performance status of 2 or higher (Table 1).

irTDs occurred in 110 patients (30.3%), with a mean time to onset of 150.5 days (range: 21–550) and a median of 114 days. Hypothyroidism was the most common presentation, observed in 92 patients (25.3%), including 30 (8.2%) who experienced a preceding hyperthyroid phase. Isolated hyperthyroidism occurred in 18 (4.9%) patients. The mean time to onset was 159.8 (range: 34–550) days for hypothyroidism and 109.8 days for hyperthyroidism (range: 21–312) (Table 2). Based on timing, 34 (30.9%) cases were classified as early-onset and 76 (69.1%) as late-onset irTDs. All events were grade 1 or 2 per NCI-CTCAE v5.0, and none led to pembrolizumab discontinuation.

Baseline characteristics were compared between patients who developed thyroid immune-related dysfunction and those who did not. No statistically significant differences were observed with respect to sex, age, smoking history, histological subtype, number of metastatic sites, PD-L1 tumor proportion score, or receipt of radiotherapy. However, thyroid immune-related dysfunction occurred more frequently in patients with good performance status (ECOG PS 0–1) (Table 3).

After a median follow-up period of 18.2 months, progression-free survival in the overall cohort reached a median of 9.8 months (95% confidence interval [CI], 7.264–12.336). With respect to treatment efficacy, best radiological responses to pembrolizumab consisted of complete response in 1.3% of patients, partial response in 32.3%, and stable disease in 38.6%. These outcomes translated into an overall response rate of 33.6% and a disease control rate of 72.2%. Occurrence of irTD was associated with more favorable responses to treatment (*p* < 0.001) (Table 4).

**Figure 1:**
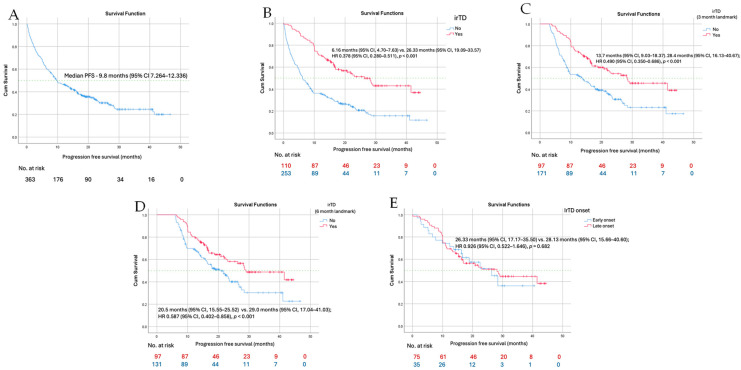
Kaplan–Meier curve of progression-free survival (PFS) for the whole cohort (**A**), patients with and without irTD in the whole cohort (**B**), at 3-month (**C**) and 6-month landmark analysis (**D**) and patient with early versus late onset of irTD (**E**).

Patients who developed immune-related thyroid dysfunction (irTD) had significantly longer progression-free survival (PFS) compared to those without irTD: 26.33 months (95% CI, 19.09–33.57) vs. 6.16 months (95% CI, 4.70–7.63); HR 0.378 (95% CI, 0.280–0.511), *p* < 0.001. To address the potential impact of immortal time bias, landmark analyses were conducted at 3 and 6 months after treatment initiation, including only patients who were progression-free at each respective time point. At the 3-month landmark, median PFS was 28.4 months (95% CI, 16.13–40.67) in patients with irTD compared to 13.7 months (95% CI, 9.03–18.37) in those without irTD; HR 0.490 (95% CI, 0.350–0.686), *p* < 0.001. At the 6-month landmark, median PFS was 29.0 months (95% CI, 17.04–41.03) in the irTD group versus 20.5 months (95% CI, 15.55–25.52) in the non-irTD group; HR 0.587 (95% CI, 0.402–0.858), *p* < 0.001. There was no significant difference in PFS between patients with early-onset and late-onset irTD: 26.33 months (95% CI, 17.17–35.50) vs. 28.13 months (95% CI, 15.66–40.60); HR 0.926 (95% CI, 0.522–1.646), *p* = 0.682 (Figure 1).

In the univariable analysis, having an ECOG PS of 0–1 and developing an irTD were both significantly associated with prolonged PFS. In the multivariable model, these factors continued to demonstrate independent prognostic value for improved PFS, while age, sex, histology, receipt of radiotherapy, CNS metastasis, smoking status, and PD-L1 expression showed no significant associations (Table 5).

## 4. Discussion

In this retrospective, single-center cohort of patients with metastatic NSCLC and high PD-L1 expression (TPS ≥ 50%) treated with pembrolizumab as first-line monotherapy, thyroid immune-related dysfunction was observed in roughly one-third of cases and was associated with a significant improvement in progression-free survival.

Overall, thyroid immune-related dysfunction developed in 30.3% of patients in our cohort, a rate that exceeds most published estimates from randomized trials and real-world studies evaluating PD-1/PD-L1 inhibitors in NSCLC. Most large series describe rates between 10% and 25%, depending on the population studied, treatment setting, and monitoring frequency.

In a PRISMA systematic review and meta-analysis of PD-1/PD-L1 inhibitors in NSCLC, Sun et al. reported an overall irAE incidence of 22% for all grades and 4% for high-grade events, with organ-specific irAEs occurring most frequently in the endocrine system and thyroid dysfunction highlighted as the most common endocrine manifestation; importantly, endocrine irAEs were predominantly low-grade, similar to our cohort [19].

In this context, the indirect comparison meta-analysis by Tartarone et al., which evaluated anti-PD-1 versus anti-PD-L1 agents in predominantly pretreated advanced NSCLC populations, provides class-level safety insights but was not designed to assess organ-specific immune-related toxicities such as thyroid dysfunction in detail. Differences in treatment line, patient selection, and adverse-event reporting frameworks limit direct comparison with real-world cohorts employing systematic laboratory surveillance [20].

Chilelli et al. reported irTD in 25.3% of 75 patients with advanced NSCLC treated with PD-1/PD-L1 monotherapy, while Thuillier et al. found a comparable incidence of 29.9% in nivolumab-treated NSCLC patients [12,21]. Similarly, Iwamoto et al. observed thyroid dysfunction in 28.2% of ICI-treated patients in a mixed-tumor cohort, and a recent systematic review by Cheung et al. encompassing over 40 studies reported a pooled incidence around 14–20% for overt thyroid dysfunction, depending on the checkpoint inhibitor used and study design [22,23].

Several factors may account for the relatively high incidence observed in our cohort. First, routine thyroid function testing every six weeks during pembrolizumab therapy likely enhanced the detection of both symptomatic and subclinical events. Second, the study population consisted exclusively of patients with PD-L1 TPS ≥ 50% receiving pembrolizumab monotherapy, a group that may experience stronger immune activation compared with those receiving combination regimens or lower PD-L1 expression levels. Third, differences in ethnic background, environmental iodine exposure, and reporting standards could also contribute to variability across studies.

Overall, the observed incidence supports the notion that thyroid dysfunction represents one of the most frequent immune-related adverse events associated with PD-1 blockade and highlights the importance of systematic thyroid monitoring during immunotherapy.

The predominance of hypothyroidism in our cohort (25.3%), frequently following a short thyrotoxic phase, mirrors the classic biphasic course of ICI-related thyroiditis. In the seminal pembrolizumab NSCLC series by Osorio et al., most thyroid events presented with early, transient thyrotoxicosis that evolved to hypothyroidism and were strongly associated with thyroid autoantibodies, supporting a destructive thyroiditis mechanism [24]. This pattern has been consistently reported across broader ICI experiences and reviews, which note that irTDs are the most common endocrine immune-related adverse events, often beginning with painless thyrotoxicosis and culminating in overt or subclinical hypothyroidism requiring long-term levothyroxine [25,26]. Importantly, consistent with our findings, most events are grade 1–2, clinically manageable, and rarely mandate immunotherapy discontinuation. Expert guidance supports continuing ICI therapy while treating hypothyroidism with levothyroxine and managing the transient hyperthyroid phase symptomatically (e.g., β-blockers) [27].

The median time to onset of thyroid dysfunction in our cohort was 114 days, consistent with previously reported intervals. In most studies, ICI-related thyroid abnormalities appear within the first 6–12 weeks of therapy but can develop at any time during treatment. Osorio et al. observed a median onset of 6 weeks for thyrotoxicosis and 10 weeks for subsequent hypothyroidism in patients receiving PD-1 inhibitors, while Kotwal et al. reported a median onset of 8 weeks for overt thyroiditis [24,28]. Similarly, other large cohorts have reported that most cases occur within the first 2–3 months of therapy, although thyroid dysfunction can develop at any point during treatment [25,29,30,31]. Collectively, these data indicate that while most thyroid irAEs develop within the first three months of immune checkpoint blockade, continued thyroid monitoring throughout treatment remains essential to detect delayed or late-onset events.

Consistent with our findings, several recent studies in NSCLC populations have shown that thyroid immune-related adverse events (irAEs) are associated with improved treatment efficacy. Chilelli et al. reported that patients who developed thyroid dysfunction during PD-1/PD-L1 therapy achieved significantly higher response rates and markedly longer PFS and OS [12]. Similarly, Cai et al. found that irTD independently predicted prolonged PFS in a large NSCLC cohort [13]. In a more recent analysis, Cerić et al. likewise demonstrated that irTD predicted significantly longer PFS among patients with advanced NSCLC receiving immune checkpoint blockade [32]. These observations echo earlier results by Wu et al., who demonstrated that thyroid dysfunction during PD-1 inhibitor therapy was linked to longer PFS [33]. Although D’Aiello et al. did not observe a statistically significant PFS improvement in a multi-ethnic NSCLC cohort, the same study noted a trend toward favorable outcomes among patients with thyroid abnormalities [14]. Taken together, these NSCLC-specific studies, alongside our results, strengthen the evidence that thyroid irAEs represent a clinically meaningful biomarker of enhanced immune activation and durable benefit from PD-1/PD-L1 blockade.

We further examined whether the timing of thyroid irAE onset influences PFS by comparing early (≤90 days) versus late (>90 days) events. In our NSCLC cohort, PFS did not differ significantly by onset category (HR 0.926; 95% CI 0.522–1.646; *p* = 0.682), suggesting that the prognostic signal of irTD may be timing-agnostic. This aligns with larger NSCLC analyses of any irAE, where the occurrence of irAEs is associated with clinical benefit but the added value of earlier versus later onset diminishes after rigorous landmark/time-dependent adjustment. For example, Yu et al. used 2-, 3-, 6-, and 9-month landmarks in NSCLC and showed the survival advantage persisted though was attenuated at later landmarks, indicating that irAE presence including irTD —rather than its exact timing—may carry the principal prognostic information [34]. Similarly, pooled atezolizumab NSCLC trials and real-world series that modeled time to irAE found consistent benefits with irAEs overall, with reduced effect sizes once timing bias was addressed [10]. Collectively, current NSCLC evidence supports irAE occurrence as a marker of benefit, while precise early-vs-late thyroid irAE stratification remains insufficiently studied; larger NSCLC datasets that explicitly pre-specify timing windows for thyroid-specific irAEs are warranted.

In our cohort, patients with poor performance status (ECOG ≥ 2) experienced significantly worse clinical outcomes, consistent with established evidence that ECOG PS is one of the strongest prognostic factors in advanced NSCLC. Multiple studies have demonstrated that NSCLC patients with impaired functional status derive less benefit from ICIs, likely reflecting limited immune reserve, reduced treatment exposure, and higher comorbidity burden [11,35,36,37].

Good ECOG PS has been associated with a higher likelihood of developing irAEs in several real-world studies of patients with NSCLC treated with ICIs [36,38,39]. Patients in good general condition appear more prone to mounting robust immune responses, which may underlie both improved therapeutic efficacy and an increased incidence of irAEs. Conversely, emerging evidence indicates that those with poor ECOG PS tend to experience fewer irAEs, including thyroid dysfunction, likely reflecting reduced immune reactivity and lower systemic inflammation. This pattern was also observed in our cohort, where patients with impaired performance status not only demonstrated worse outcomes but also a lower incidence of irTD, supporting the notion that the development of immune-related toxicity may serve as an easily detectable and affordable surrogate for effective immune activation.

## 5. Limitations

This study has several limitations related to its retrospective, single-center design. The cohort was restricted to patients with PD-L1 TPS ≥ 50% treated with first-line pembrolizumab monotherapy, reflecting reimbursement constraints at our institution, where this was the only reimbursed first-line option during the study period. Although this limits generalizability to broader NSCLC populations and combination regimens, it ensured a homogeneous treatment cohort and reduced therapeutic confounding. Additionally, molecular testing was limited to EGFR and ALK alterations, as broader genomic profiling was not routinely available in Serbia during the study period, which may have led to incomplete exclusion of rare oncogenic drivers.

Thyroid function testing was performed at baseline and approximately every six weeks in accordance with guideline-based clinical practice. However, this interval may have missed transient or asymptomatic thyrotoxic phases and introduces some imprecision in onset timing. While time-varying covariate modeling and landmark analyses were used to mitigate immortal-time bias, residual confounding related to treatment duration and surveillance intensity cannot be fully excluded.

Several potentially relevant baseline variables were unavailable, including systemic corticosteroid exposure, autoimmune disease history, inflammatory markers, antibiotic use, comorbidity burden, and detailed characterization of metastatic burden and radiotherapy timing. Baseline TSH levels were available for all patients, and individuals with abnormal thyroid function or known thyroid disease were excluded; however, thyroid autoantibodies were not routinely assessed, limiting differentiation between ICI-induced thyroid dysfunction and latent autoimmune predisposition.

Finally, overall survival data were immature, necessitating progression-free survival as the primary endpoint. As a real-world study conducted at a single academic center, external validity may be limited. Despite these constraints, the large, clinically homogeneous cohort and robust analytical approach support the clinical relevance of our findings, which should be considered hypothesis-generating and warrant prospective validation.

## 6. Conclusions

In this real-world cohort of patients with metastatic, non-oncogene-addicted NSCLC and high PD-L1 expression treated with first-line pembrolizumab, the development of irTD was common and independently associated with significantly longer progression-free survival. In addition, good performance status (ECOG 0–1) was an important determinant of improved outcomes, underscoring the role of baseline patient fitness in treatment efficacy. These findings support the growing evidence that irTD may serve as a surrogate marker of effective immune activation and favorable response to immune checkpoint inhibitors. Given its frequency, ease of detection, and clinical manageability, thyroid dysfunction represents a practical candidate biomarker for monitoring immunotherapy efficacy. Prospective studies with mature overall survival data and comprehensive endocrine assessment are warranted to validate the predictive value of irTD and clarify its underlying mechanisms.

## Figures and Tables

**Table 1:** Demographic data of included patients.

N = 363	N (%)
Mean age at treatment start (range) [years]	65.77 (40–86)
Sex	
Male	216 (59.5)
Female	147 (40.5)
Smoking status	
Current	254 (63.9)
Ex smoker	84 (23.1)
Non-smoker	25 (9.5)
ECOG PS	
0–1	266 (74.3)
≥2	97 (26.7)
Histological diagnosis	
Adenocarcinoma	247 (68.0)
Squamous cell carcinoma	86 (23.7)
Other (NOS)	30 (8.3)
PD-L1 TPS	
50–79%	206 (56.7)
80–100%	157 (43.3)
Radiotherapy during treatment	
Yes	85 (23.5)
No	278 (76.5)
Number of metastatic sites	
Mean	1.37
3<	339 (93.3)
3≥	24 (6.7)
CNS metastasis at baseline	
Yes	75 (20.6)
No	288 (79.4)
Immune related thyroid disorders	
Yes	110 (30.3)
No	253 (69.6)
Best response to pembrolizumab	
^α^PD	101 (27.8)
^β^SD	140 (38.6)
^δ^PR	117 (32.3)
^μ^CR	5 (1.3)
Real world °DCR	72.2%
Real world ^ö^ORR	33.6%
Median PFS (95% Confidence Interval) [months]	9.8 (95% CI 7.264–12.336)

^α^PD—progressive disease; ^β^SD—stable disease ^δ^PR—partial response; ^μ^CR—complete response; °DCR—disease control rate; ^ö^ORR—overall response rate.

**Table 2:** Types and median times of onset of irTD.

irTD	N = 110 (%)	Mean Time of Onset—150.5 (Range: 21–550; Days)
Hypothyroidism	92 (83.6%)	159.8 (range: 34–550)
Preceded by a hyperthyroid phase	30 (27.2%)	
Hyperthyroidism	18 (4.9%)	109.8 (range: 21–312)

**Table 3:** Characteristics of patients that developed irTD and those that did not.

	Patients with irTD (110)	Patients Without irTD (253)	*p* Value
**Age**			0.41
<70 years	56 (51%)	117 (46%)	
≥70 years	54 (49%)	136 (54%)	
**Sex**			0.55
Male	66 (60%)	160 (63%)	
Female	44 (40%)	93 (37%)	
**Histology**			0.41
non-squamous	87 (79%)	190 (75%)	
squamous	23 (21%)	63 (25%)	
**Smoking status**			0.84
Current or former	102 (92%)	236 (93%)	
never-smoker	8 (8%)	17 (7%)	
**PD-L1 status**			0.56
50–79%	63 (57%)	153 (60%)	
80–100%	47 (43%)	100 (40%)	
**ECOG PS**			0.04
0–1	89 (81%)	177 (70%)	
2	21 (19%)	76 (30%)	
**Radiotherapy**			0.84
Yes	25 (47.8%)	60 (35.4%)	
No	85 (52.2%)	193 (64.6%)	
**CNS metastasis**			0.95
Yes	22 (20%)	53 (21%)	
No	88 (80%)	200 (79%)	

**Table 4:** Response to treatment among patients that have experienced an irTD.

	irTD Yes—N [110] (%) No—N [253] (%)	
CR	2 (1.8%)	3 (1.1%)
PR	57 (51.8%)	60 (23.6%)
SD	41 (36.4%)	100 (39.4%)
PD	10 (9%)	90 (35.9%)

**Table 5:** Univariable and multivariable regression analysis.

	Univariate Regression Analysis			Multivariate Regression Analysis		
	HR	95% CI	*p*	HR	95% CI	*p*
Age (<70 years vs. ≥70 years)	0.991	0.562–1.748	0.974			
Sex (male vs. female)	1.083	0.676–1.735	0.741			
Histology (non-squamous vs. squamous)	1.056	0.674–1.653	0.813			
Radiotherapy (Yes vs. No)	0.804	0.597–1.082	0.150			
Smoking status (ever vs. never-smoker)	1.312	0.837–2.057	0.237			
PD-L1 (50–80% vs. 80–100%)	0.917	0.470–1.787	0.798			
CNS metastasis (Yes vs. No)	0.985	0.717–1.353	0.926			
ECOG PS (2 vs. 1–2)	7.744	4.440–13.508	<0.001	7.124	4.061–12.497	<0.001
irTD (yes vs. no)	0.365	0.195–0.681	0.002	0.457	0.242–0.863	0.016

## Data Availability

The dataset generated and analyzed during the current study is not publicly available due to privacy regulations and consent restrictions but is available from the corresponding author upon reasonable request. All data shared will be de-identified in accordance with ethical guidelines.
